# Astragaloside IV alleviates renal fibrosis by inhibiting renal tubular epithelial cell pyroptosis induced by urotensin II through regulating the cAMP/PKA signaling pathway

**DOI:** 10.1371/journal.pone.0304365

**Published:** 2024-05-31

**Authors:** Lin Zhang, Wenyuan Liu, Sufen Li, Jinjing Wang, Dalin Sun, Hui Li, Ziyuan Zhang, Yaling Hu, Jingai Fang

**Affiliations:** 1 Shanxi Medical University, Taiyuan, Shanxi Province, China; 2 Department of Prevention Care, Cardiovascular Hospital of Shanxi Medical University, Taiyuan, Shanxi Province, China; 3 Department of Nephrology, First Hospital of Shanxi Medical University, Taiyuan, Shanxi Province, China; University of Hyderabad, INDIA

## Abstract

**Objective:**

To explore the molecular mechanism of Astragaloside IV (AS-IV) in alleviating renal fibrosis by inhibiting Urotensin II-induced pyroptosis and epithelial-mesenchymal transition of renal tubular epithelial cells.

**Methods:**

Forty SD rats were randomly divided into control group without operation: gavage with 5ml/kg/d water for injection and UUO model group: gavage with 5ml/kg/d water for injection; UUO+ AS-IV group (gavage with AS-IV 20mg/kg/d; and UUO+ losartan potassium group (gavage with losartan potassium 10.3mg/kg/d, with 10 rats in each group. After 2 weeks, Kidney pathology, serum Urotensin II, and cAMP concentration were detected, and the expressions of NLRP3, GSDMD-N, Caspase-1, and IL-1β were detected by immunohistochemistry. Rat renal tubular epithelial cells were cultured in vitro, and different concentrations of Urotensin II were used to intervene for 24h and 48h. Cell proliferation activity was detected using the CCK8 assay. Suitable concentrations of Urotensin II and intervention time were selected, and Urotensin II receptor antagonist (SB-611812), inhibitor of PKA(H-89), and AS-IV (15ug/ml) were simultaneously administered. After 24 hours, cells and cell supernatants from each group were collected. The cAMP concentration was detected using the ELISA kit, and the expression of PKA, α-SMA, FN, IL-1β, NLRP3, GSDMD-N, and Caspase-1 was detected using cell immunofluorescence, Western blotting, and RT-PCR.

**Results:**

Renal tissue of UUO rats showed renal interstitial infiltration, tubule dilation and atrophy, renal interstitial collagen fiber hyperplasia, and serum Urotensin II and cAMP concentrations were significantly higher than those in the sham operation group (*p* <0.05). AS-IV and losartan potassium intervention could alleviate renal pathological changes, and decrease serum Urotensin II, cAMP concentration levels, and the expressions of NLRP3, GSDMD-N, Caspase-1, and IL-1β in renal tissues (*p* <0.05). Urotensin II at a concentration of 10^−8^ mol/L could lead to the decrease of cell proliferation, (*p*<0.05). Compared with the normal group, the cAMP level and the PKA expression were significantly increased (*p*<0.05). After intervention with AS-IV and Urotensin II receptor antagonist, the cAMP level and the expression of PKA were remarkably decreased (p<0.05). Compared with the normal group, the expression of IL-1β, NLRP3, GSDMD-N, and Caspase-1 in the Urotensin II group was increased (*p*<0.05), which decreased in the AS-IV and H-89 groups.

**Conclusion:**

AS-IV can alleviate renal fibrosis by inhibiting Urotensin II-induced pyroptosis of renal tubular epithelial cells by regulating the cAMP/PKA signaling pathway.

## 1. Introduction

Chronic kidney disease (CKD) has high morbidity and has become a serious problem affecting global public health. Renal fibrosis, characterized by glomerular sclerosis and renal interstitial fibrosis, plays a core role in promoting the progression of CKD to end-stage [1,2]. Its occurrence involves various pathophysiological mechanisms such as inflammation, epithelial-mesenchymal transformation, and oxidative stress [3–5]. Renal interstitial fibrosis (RIF) caused by tubular atrophy and tubular epithelial cell injury is the main cause and common pathological process, leading to chronic renal failure and end-stage renal disease, and tubular epithelial cell injury is the key factor and central link of RIF [6]. Epithelial interstitial transformation (EMT) plays an important role in RIF [7], and its mechanism and influencing factors include the TGF-β1 pathway [8] and inflammatory factors such as NF-κB activation [9]. After the injury of renal tubular epithelial cells, a large number of pro-inflammatory factors are released and inflammatory cells are recruited, further aggravating kidney injury [10]. A recent study suggests that the release of pro-inflammatory factors is mainly dependent on cell pyroptosis, which is an important mechanism of renal fibrosis [11]. Inhibition of renal tubular epithelial cell pyroptosis is a potential way to delay RIF.

Urotensin II(UII) is a novel vasoactive peptide. Studies have confirmed [12] that UII can induce EMT in renal tubular epithelial cells, but the specific mechanism remains unclear. Recently, studies have found that the expression of UII and its receptor increases significantly with the progression of CKD, and blocking UII can delay the progression of renal fibrosis [13]. Recent studies have shown that UII can induce pyroptosis by regulating the NLRP3-caspase1 pathway, suggesting that UII may be involved in the pathogenesis of renal tubular epithelial cell EMT and renal fibrosis by inducing pyroptosis [14,15]. Previous studies have shown that GPR14, the specific receptor of UII, can regulate the cAMP/PKA signaling pathway to promote myocardial fibrosis after activation [16], and the cAMP/PKA signaling pathway is also involved in regulating the fibrosis process of other tissues and organs, such as kidney and liver [17]. Recent studies have found that PKA phosphorylation can further induce NLRP3 phosphorylation and ubiquitination, thereby blocking NLRP3 inflammasome-dependent inflammation and cell pyroptosis [18,19]. Based on the above studies, it is concluded that UII may participate in NLRP3 inflammasome-dependent cell pyroptosis through the regulation of the cAMP/PKA signaling pathway.

Astragalus, a traditional Chinese medicine, is widely used in the treatment of kidney disease. Clinical pharmacological studies [20,21] have shown that Astragalus has an anti-renal fibrosis effect, in which the active ingredient AS-IV can inhibit inflammation and epithelial-mesenchymal transformation, reduce extracellular matrix accumulation and inflammatory cell infiltration in the kidney tissue of UUO-induced renal fibrosis model, and thus inhibit the progression of renal fibrosis. Previous studies of our research group have found that Astragalus delays the process of renal interstitial fibrosis in rats with chronic overload stress by reducing the overexpression of UⅡ and collagen Ⅰ and Ⅲ in the renal interstitial, and thus plays a protective role in the kidney [22], but its specific mechanism is not yet clear. The purpose of this study is to investigate the mechanism of UII in renal tubular epithelial cell pyroptosis and the mechanism of AS-IV in improving renal tubular epithelial pyroptosis and inhibiting renal fibrosis by using UII intervention in vitro.

## 2. Materials and methods

### 2.1 Experimental materials

Rat renal tubular epithelial cells (NRK-52E) were purchased from the cell bank of the Chinese Academy of Science. UII was purchased from Wuhan Yunclon Co., LTD. UII receptor antagonist SB-611812, AS-IV, and PKA inhibitor H-89 were purchased from Shanghai MCE Company. Losartan potassium was purchased from Merck. RNA Extraction Kit and qPCR Kit were purchased from Promega Biotechnology LTD. BCA Kit was purchased from Shanghai Biyuntian Biotechnology Co., LTD. The cAMP ELISA kit was purchased from Shanghai Jianglai Biotechnology Co., LTD. The Urotensin II kit (ELISA) was purchased from Rising Biotech. PKA antibodies, Col-Ⅰ antibodies, FN antibodies, and α-SMA antibodies are bought from Proteintech LTD. IL-1β antibody, NLRP3 antibody, and Caspase-1 antibody are ordered from Beijing Bioss Company. GSDMD-N antibody and FITC labeled fluorescent secondary antibody were purchased from Zhejiang Hua ’a Biological Co., LTD. Horseradish peroxidase-labeled secondary antibody was bought from Wuhan Bode Co., LTD.

### 2.2 Animal experiments

Forty SPF-graded SD rats with body weight (180±10) g were selected and randomly divided into sham operation group, UUO model group, AS-IV group, and losartan potassium group, with 10 rats in each group. The operation was performed after 1 week of adaptive feeding, and the rats were fasted for 12 hours before the operation, fixed on the operating table, and anesthetized with 350 mg/kg intraperitoneal injection of 10% chloral hydrate. The surgical area was disinfected after hair clipping, and each layer of tissue was separated from the left abdominal incision. The left ureter was lapped near the renal portal and at both ends of the lower ureter, and the abdominal wall and skin were sutured. After the modeling was successful, the sham operation group and UUO model group were given 5ml/kg/d of water for injection, the AS-IV group was given 40mg/kg/d of AS-IV, and the losartan potassium group was given 10.3mg/kg/d of losartan potassium. After 2 weeks of intervention, the rats were killed and serum and kidney tissue specimens were collected. The experiment was approved by the Animal Ethics Committee of Shanxi Medical University (SYXK2019-0007).

### 2.3 Renal pathology

Renal cortical tissue samples were collected, fixed with paraformaldehyde embedded in paraffin, and then stained by HE and Masson. The pathological changes were observed under the optical microscope.

### 2.4 UII concentration determination

According to the procedures of the kit, serum samples of rats in each group were added to 96-well plates, with 3 replicated wells. After the samples were added, 100 μL detection antibodies labeled with horseradish peroxidase (HRP) were added, the reaction plate was covered by the sealing plate membrane, and incubated in the incubator for 60 min in the dark. Discard the liquid, add each hole with washing liquid, aged for 20 s, add substrate mixture 100 μL, cover the reaction plate with sealing plate film, and incubate in the incubator for 15 min in the dark. The reaction was terminated by adding a termination solution of 50 μL, the OD values of each hole were read and the corresponding concentration was calculated on the enzyme label with the wavelength of 450nm.

### 2.5 Immunohistochemical detection

Kidney tissues of each group were taken, paraffin-embedded, sliced, dehydrated, and repaired with EDTA antigen repair solution. Added 5% BSA (1:200) diluted primary antibody (IL-1β antibody, bs-0812R; NLRP3 antibody, bs-10021R; and Caspase-1 antibody, bs-0169R; Beijing Bioss Company. GSDMD-N antibody, ER1901-37, Zhejiang Hua ’a Biological Co., LTD.). Aged for 2 h at room temperature; Added secondary antibody (BA1055, Wuhan Bode Co., LTD.1:50), Aged at room temperature for 30min, rinsed with PBS for 3 times; DAB color development for 10min; Hematoxylin was dyed for 30 s, rinsed with tap water, differentiated with 1% hydrochloric acid alcohol for 3 s, rinsed with tap water for 15 minutes; and then dehydrated and sealed with neutral gum.

### 2.6 Cell culture

The frozen rat renal tubular epithelial cells (NRK-52E) were removed from liquid nitrogen, quickly melted in a water bath at 37°C, centrifuged at 1000r/min for 5min, and then added to the culturing medium and cultured in an incubator at 37°C with 5% CO_2_.

### 2.7 CCK8 detection

The effects of UII at different concentrations (10^−4^ uM, 10^−3^ uM, 10^−2^ uM, 10^−1^ uM) on the proliferation of NRK-52E cells at different time points (24h, 48h) were detected. When the cell adhesion growth reached about 90%, the cells were cultured in 96-well plates and cultured in cell incubators for 24 h. After observing that the cells were in the adhesion state, different concentrations of UII 100 μL were added to each well and cultured in cell incubators for 24 h. 10 μL cell proliferation detection reagents were added to each well and incubated in cell incubators for 1–4 h. OD values of each group were measured at 450 nm with an enzyme marker. The appropriate concentration and intervention time were selected for follow-up experiments.

### 2.8 Cell experiment grouping

Control group (DMEM medium, N); Urotensin II group (DMEM medium + UII, UII); Urotensin II receptor antagonist group (DMEM medium +10^−2^ μM UII +1 μM SB-611812, UII+SB); PKA inhibitor group (DMEM medium+10^−2^ μM UII+10 μM H-89, UII+H-89); AS-IV group (DMEM medium +10^−2^ μM UII+15ug/ml AS-IV, UII+AS-IV). The cells were collected from each group after 24 hours of intervention.

### 2.9 Cellular immunofluorescence

After the intervention, the medium was discarded, washed with PBS 3 times, and fixed at room temperature with 4% paraformaldehyde for 30 minutes. Cells were perforated with 0.5% Triton X-100 at room temperature for 5min and washed 3 times with PBS. Adding 3% BSA and blocking at room temperature for 30min; Adding 3% BSA diluted primary antibody (FN antibodies, 15613-1-AP; α-SMA antibodies, 14395-1-AP; Proteintech LTD.1:500) and put it in a wet box and incubate overnight in a refrigerator at 4°C. Washed with PBS 3 times, added FITC fluorescence-labeled secondary antibody (HA1106; Zhejiang Hua ’a Biological Co., LTD. 1:50), and incubated in a wet box at room temperature for 2 h away from light; PBS was used to wash them 3 times and DAPI was added and incubated for 5min in dark light. After washing with PBS 3 times, the sealing liquid of the anti-fluorescence quencher was used to seal the samples and observed under a confocal microscope. The parameter of red wavelength 647 nm was selected.

### 2.10 cAMP concentration detection

The cell supernatant of each group and the Serum of rats in each group were collected and cAMP concentration was detected by EILSA kit (JL10117; Shanghai Jianglai Biotechnology Co., LTD.). The sample was added 50 μl into the corresponding 96-well plate, with 3 repeated wells in each group. The blank well was added with 50 μL general diluent and then each well with 50 μL antibody working liquid was added. The sealing plate was covered and incubated at 37°C for 1 h. After discarding the liquid, wash 3 times, add 100 μL of enzyme-conjugated working liquid to each well, cover the sealing plate film, and incubate at 37°C for 30 min. After discarding the liquid, wash 5 times, adding 90 μL substrate (TMB), covering the sealing plate film, and warming at 37°C for 15 min. Adding 50 μL of the termination solution, and immediately measuring the OD value of each well at 450 nm wavelength to calculate the sample concentration.

### 2.11 Western blotting

The cells of each group were collected, and put on ice at 4°C for 1h after adding RIPA lysate and PMSF, and the supernatant was collected and stored at -80°C after centrifugation. The protein concentration was determined by the BCA method. The appropriate amount of kidney tissue was ground and centrifuged. The supernatant was collected and stored at -80°C. Protein concentration was determined by the BCA method, and then boiled in boiling water for 10min after loading buffer was added and then stored at -20°C in the refrigerator. The protein samples were subjected to 90V constant pressure electrophoresis for 30 min and 120V electrophoresis for 1.5h. The membrane translated at 4°C. 230 mA constant current electrophoresis was performed for 60min. The membrane was blocked with 5% milk at room temperature for 1h. The diluted primary antibody (PKA antibodies, 27398-1-AP; Col-Ⅰ antibodies, 14695-1-AP; FN antibodies, 15613-1-AP; α-SMA antibodies, 14395-1-AP; Proteintech LTD. IL-1β antibody, bs-0812R; NLRP3 antibody, bs-10021R; and Caspase-1 antibody, bs-0169R; Beijing Bioss Company. GSDMD-N antibody, ER1901-37, Zhejiang Hua ’a Biological Co., LTD.1:1000) were added to the membrane and incubated at 4°C overnight. TBST was used to wash it 3 times for 10 minutes each time. A diluted secondary antibody (BA1055, Wuhan Bode Co., LTD.1:50) was added and incubated at room temperature for 1–2 h, then TBST was used to wash it 3 times for 10 min each time. Exposure after adding ECL luminescent solution.

### 2.12 RT-PCR

The primer sequences are shown in Table 1. The cells of each group were collected and total RNA was extracted by Trizol method. An appropriate amount of kidney tissue was taken and added to Trizol. The supernatant was collected by centrifugation after grinding in a tissue homogenizer. Total RNA was extracted and the concentration was determined and stored in a -80°C refrigerator. cDNA was synthesized by reverse transcription kit and stored in a -20°C refrigerator. The enzyme-free EP tube was added with Master Mix 2X, cDNA template, upstream primer, downstream primer, and DEPC H2O. The sample was gently mixed and centrifuged. The PCR procedure was set as follows: pre-denaturation 95°C×10 min→ denaturation 95°C× 15s→ annealing 60°C×1 min. Quantitative RT-PCR was used to amplify and detect CT values, and the results were calculated using 2^-ΔΔCT^ to obtain mRNA expression levels.

**Table 1:** Primer pair sequence of target genes.

Gene Name		Gene Sequence (5’→3’)
NLRP3	Forward	TCTCTGCATGCCGTATCTGG
	Reverse	ACGGCGTTAGCAGAAATCCA
Caspase-1	Forward	CACGAGACCTGTGCGATCAT
	Reverse	GCGCCACCTTCTTTGTTCAG
IL-1β	Forward	CACTACAGGCTCCGAGATGAACAAC
	Reverse	TGTCGTTGCTTGGTTCTCCTTGTAC
GSDMD	Forward	AAGATCGTGGATCATGCCGT
	Reverse	CGGGGTTTCCAGAACCATGA
α-SMA	Forward	GGAGATGGCGTGACTCACAA
	Reverse	CGCTCAGCAGTAGTCACGAA
FN	Forward	GGATCCCCTCCCAGAGAAGT
	Reverse	GGGTGTGGAAGGGTAACCAG
β-actin	Forward	CACGATGGAGGGGCCGGACTCATC
	Reverse	TAAAGACCTCTATGCCAACACAGT

### 2.13 Statistical methods

SPSS 22.0 statistical software was used for analysis. If the measurement data followed a normal distribution, it was expressed as mean ± standard deviation. One-way ANOVA was used for inter-group comparisons, and the LDS-t test was used for multiple comparisons. For non-normal distribution or uneven variance, a non-parametric test was used for multiple independent samples, and an extended t-test was used for multiple comparisons. *P* < 0.05 was considered statistically significant.

## 3. Results

### 3.1 Pathological changes of renal tissue of rats in each group

**Fig 1:**
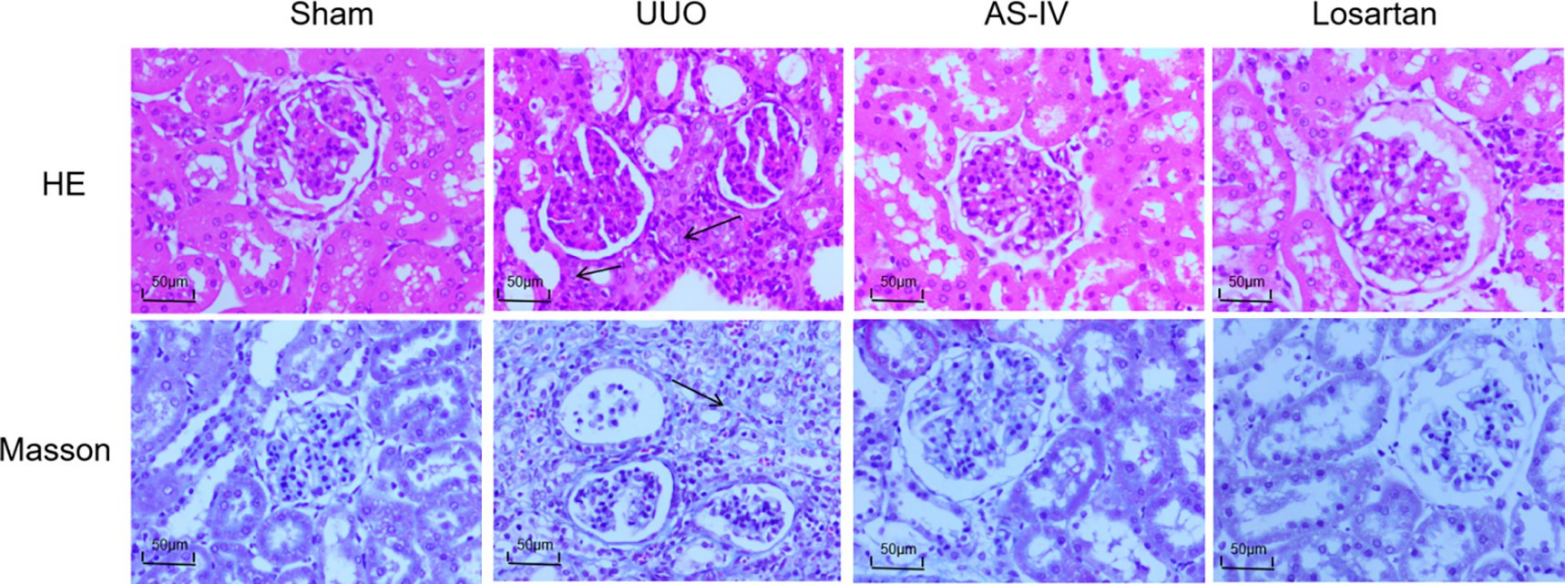
Pathological changes of renal tissue in each group. The black arrow indicates a massive infiltration of inflammatory cells in the renal interstitium, dilation of renal tubules, and proliferation of collagen fibers (scale bar: 100 μm).

HE staining results showed that the morphology of glomeruli and renal tubules in the sham operation group was normal, while the renal tubules in the UUO group were disordered, some renal tubules were dilated, epithelial cells were shed, and a large number of inflammatory cells infiltrated the renal interstitium. The number of renal interstitial inflammatory cell infiltration in the AS-IV group and losartan potassium group was less than that in the UUO group, and tubule dilation and epithelial cell shedding were less than that in the UUO group, suggesting that AS-IV could improve renal fibrosis in UUO rats. Masson staining showed renal interstitial collagen fiber hyperplasia in the UUO group, while pathological changes in the AS-IV group and Losartan potassium group were alleviated (Fig 1).

### 3.2 Effect of AS-IV on cAMP and UII concentration of UUO rats

**Fig 2:**
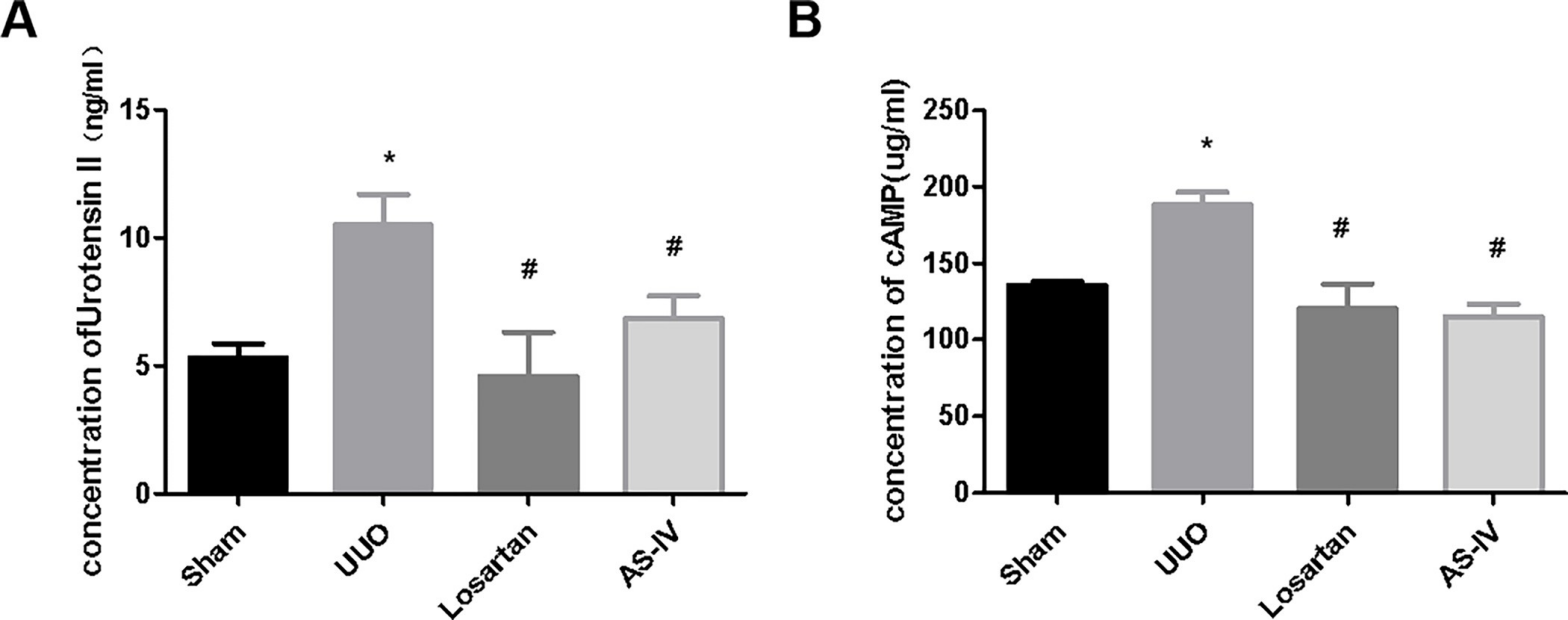
Effects of AS-IV on concentration of cAMP and UII in UUO rats. (A) Effects of AS-IV on UII concentration in UUO rats. (B) The effect of AS-IV on cAMP concentration in UUO rats. * means *p*< 0.05 compared with group Sham, # means *p*<0.05 compared with group UUO. The experiment was repeated three times.

The results showed that the serum concentrations of cAMP and UII in the UUO group were significantly higher than those in the sham operation group (*p* <0.05), and the serum concentrations of cAMP and UII in the AS-IV group and losartan potassium group were lower than those in UUO group (*p* < 0.05). It was suggested that cAMP and UII were involved in renal fibrosis in UUO rats, and AS-IV had an alleviating effect on renal fibrosis (Fig 2).

### 3.3 Effect of AS-IV on pyroptosis of renal tissue in UUO rats

**Fig 3:**
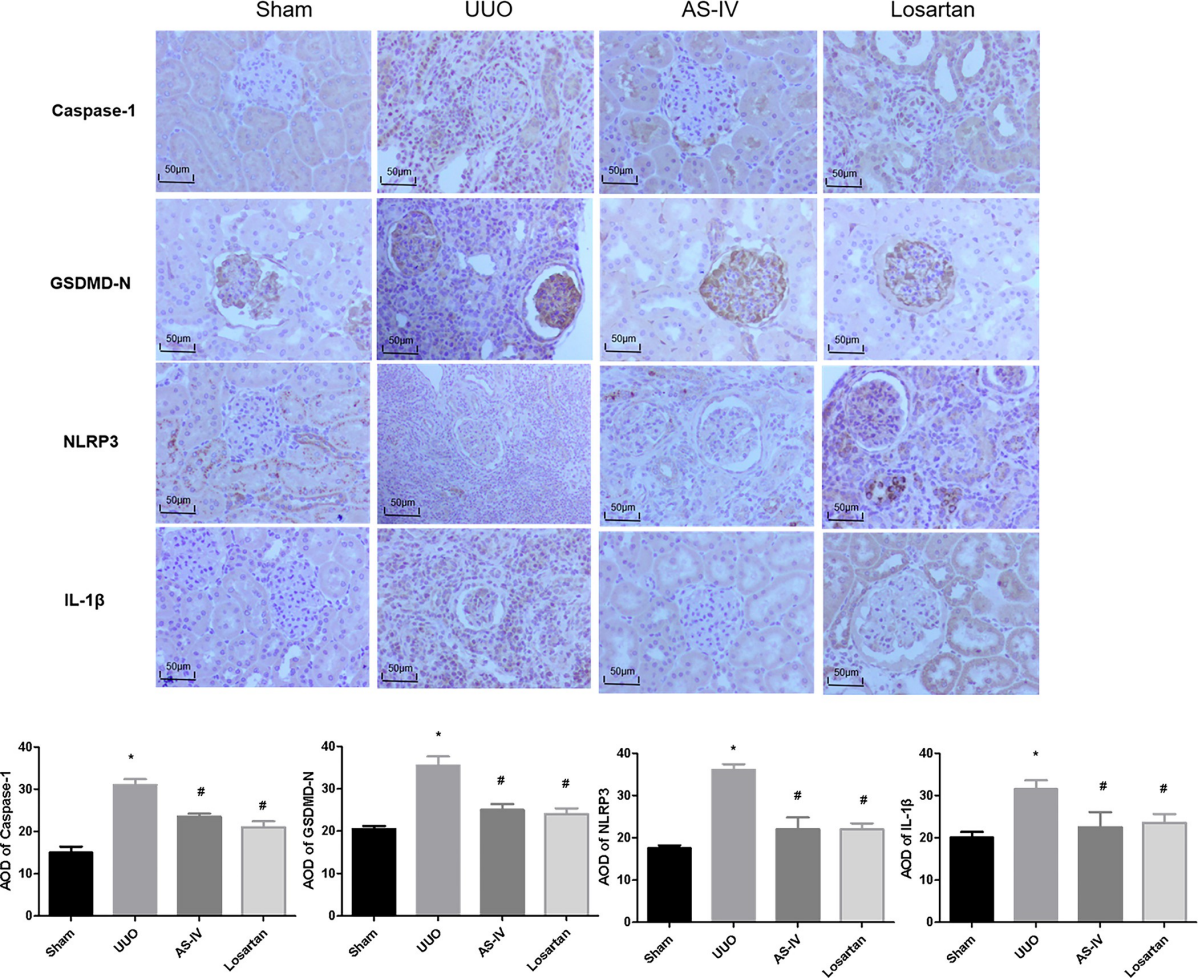
Expression of NLRP3, GSDMD-N, Caspase-1, and IL-1β in renal tissues of rats in each group. * means *p* < 0.05 compared with group Sham, # means *p*<0.05 compared with group UUO (scale bar: 50 μm). The experiment was repeated three times.

Immunohistochemical results showed that IL-1β, NLRP3, and Caspase-1 were mainly expressed in the cytoplasm of tubule epithelial cells, and GSDMD-N was expressed in the membrane of tubule epithelial cells. The expressions of IL-1β, NLRP3, GSDMD-N, and Caspase-1 in the UUO group were higher than those in the sham operation group (*p* < 0.05), while those in the AS-IV group and Losartan potassium group were lower than those in UUO group (*p* <0.05) (Fig 3).

### 3.4 Effects of UII on the proliferation of renal tubular epithelial cells

**Fig 4:**
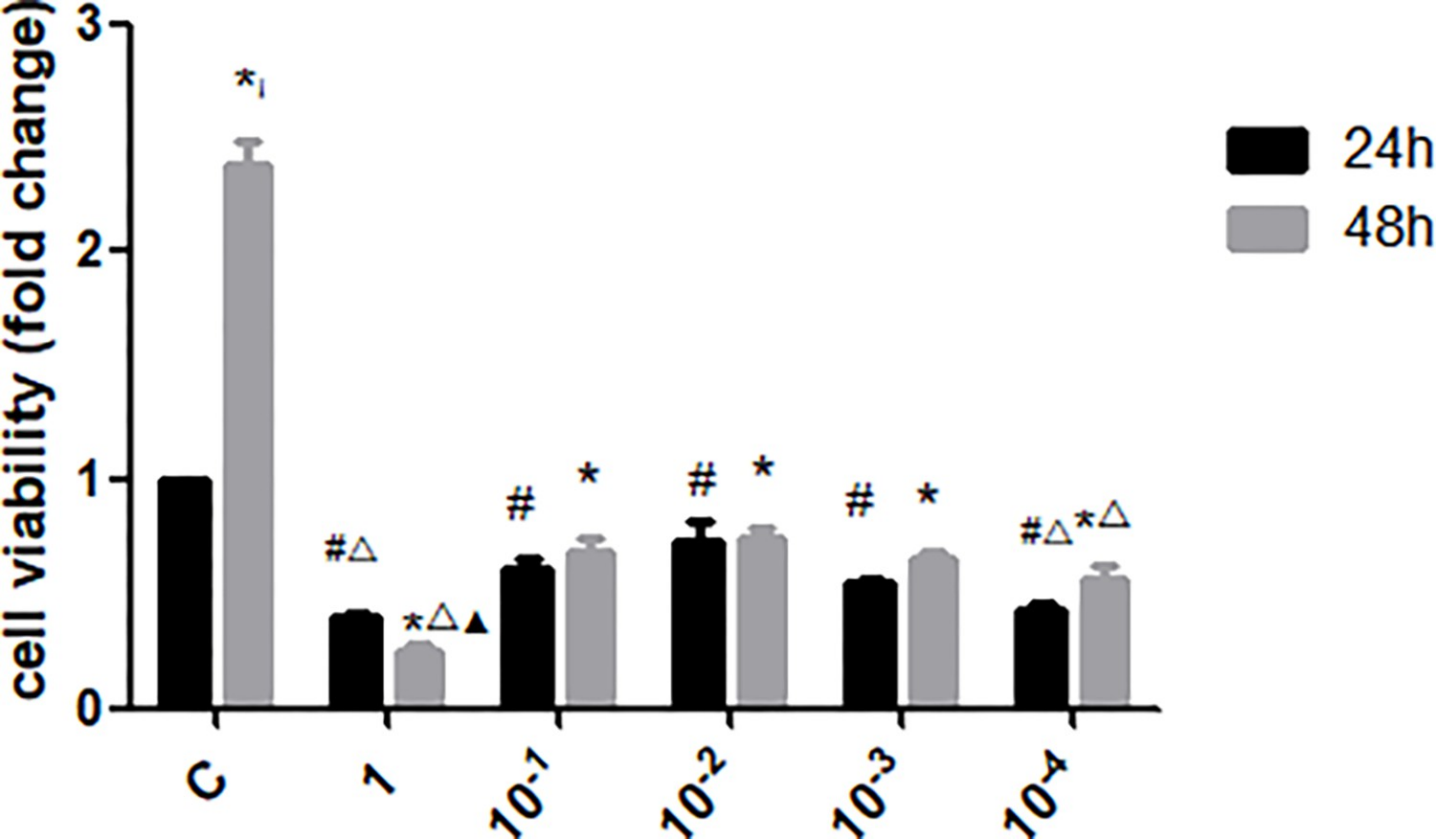
Effects of UII on the proliferation of renal tubular epithelial cells. * indicates *p*<0.05 compared with group C; # indicates *p*<0.05 compared with group C; △ indicates *p*<0.05 compared with 10^−2^ uM; ▲ indicates *p*<0.05 of the comparison between 48h and 24h.The experiment was repeated three times.

NRK-52E cells were cultured *in vitro*. The effects of UII at different concentrations (10^−4^ uM, 10^−3^ uM, 10^−2^ uM, 10^−1^ uM, 1 uM) on the viability of NRK-52E cells at different time points (24h, 48h) were detected by CCK8 kit. The results showed that UII could lead to decreased cell viability, and the effect of UII on cell viability at 10^−2^ uM, was lower than that at 10^−4^ uM and 1 uM (*p* <0.05), but higher than that at 10^−3^ uM and 10^−1^ uM (Fig 4). However, there was no significant difference (Fig 1, *p*>0.05). In the UII group, the cell viability after UII treatment for 48 h was decreased compared with the control group (*p*<0.05), and the cell viability was similar between 24h and 48h at 10^−2^ uM concentration (Fig 4). Therefore, 10^−2^ uM UII intervention was selected for follow-up experiments

### 3.5 Effects of AS-IV on cAMP/PKA signaling pathway in renal tubular epithelial cells after UII intervention

**Fig 5:**
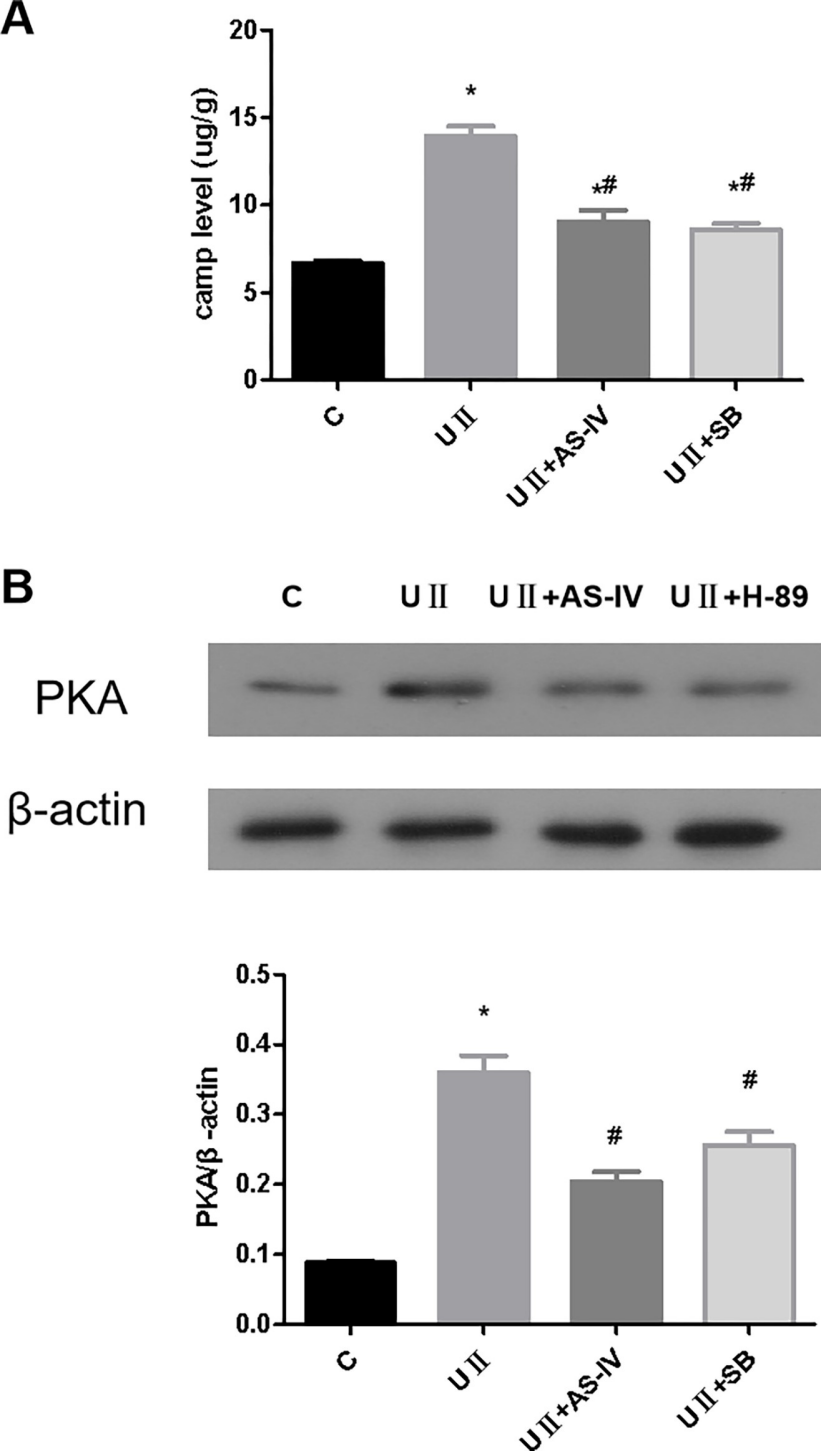
Effects of AS-IV on cAMP/PKA signaling pathway in renal tubular epithelial cells after UII intervention. (A) Effects of AS-IV on cAMP concentration in renal tubular epithelial cells after UII intervention. (B) The effect of AS-IV on PKA protein expression in renal tubular epithelial cells after UII intervention. * means *p*<0.05 compared with group C, # means *p*<0.05 compared with group UⅡ. The experiment was repeated three times.

The results showed that the cAMP level and PKA expression level in the UII intervention group were higher than those in the control group (Fig 2, *p*<0.05). As shown in Fig 5, after intervention with AS-IV and UII receptor antagonists, cAMP levels and PKA expression decreased (*p*<0.05).

### 3.6 Effect of AS-IV on pyroptosis of renal tubular epithelial cells induced by UII

**Fig 6:**
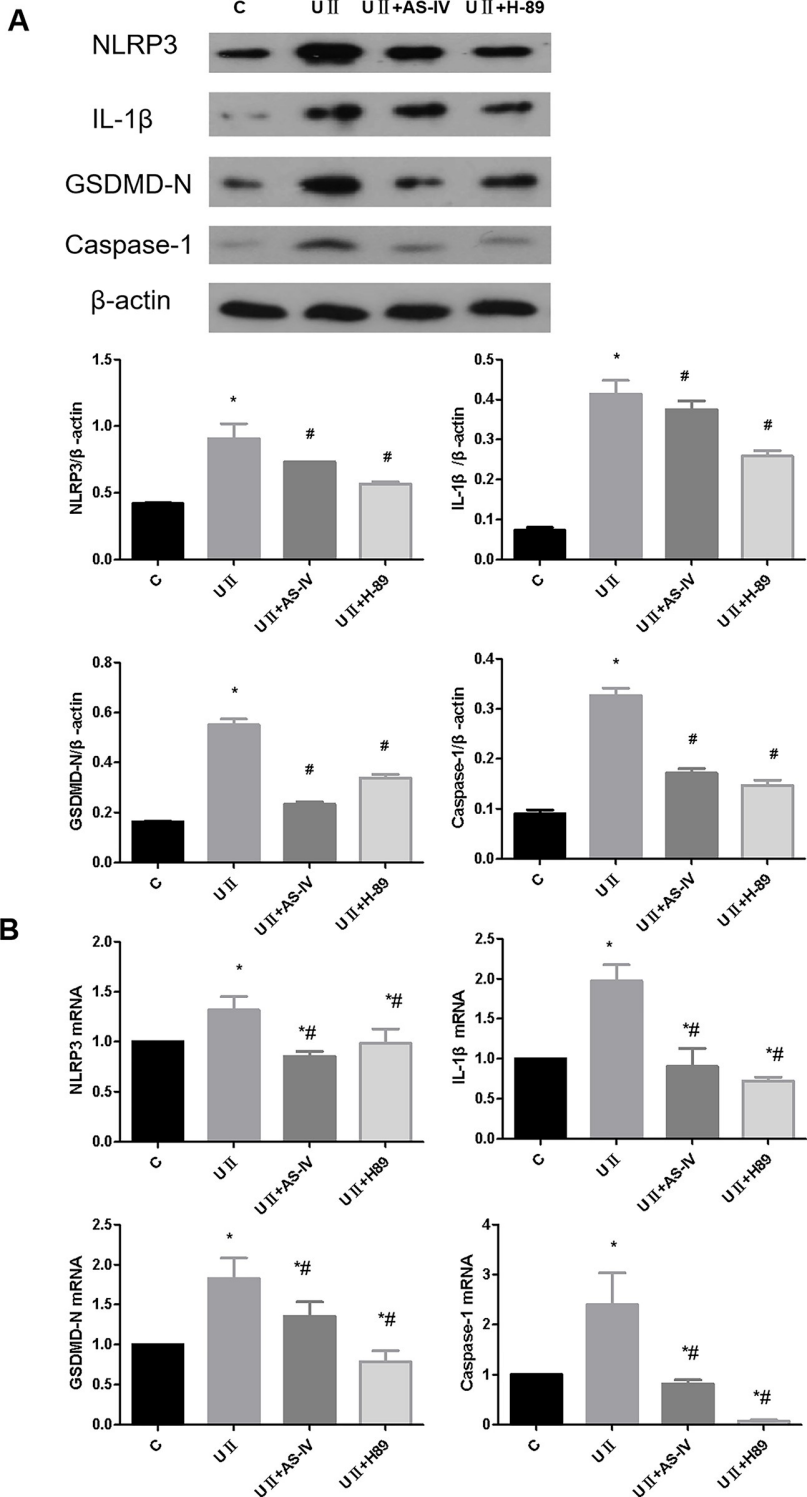
Effect of AS-IV on UII-induced renal tubular epithelial cells pyroptosis. (A) Effect of AS-IV on the expression of NRK-52E pyroptosis proteins induced by UII. (B) Effect of AS-IV on mRNA expression of pyroptosis-related factors induced by UII in NRK-52E cells. * means *p*<0.05 compared with group C, # means *p*<0.05 compared with group UII. The experiment was repeated three times.

The results showed that UII could significantly upregulate the protein and mRNA expression of IL-1β, NLRP3, GSDMD-N as well as Caspase-1 in NRK-52E cells (Fig 3, *p* <0.05). After the intervention of AS-IV and PKA inhibitors, the mRNA expression of NRK-52E cells was increased. After treatment with AS-IV and PKA inhibitors, the protein and mRNA expressions of IL-1β, NLRP3, GSDMD-N, and Caspase-1 in NRK-52E cells were significantly decreased compared with those in the UⅡ group (Fig 6, *p* <0.05). These results suggest that UII can induce pyroptosis of renal tubular epithelial cells, while AS-IV can inhibit UII-induced pyroptosis.

### 3.7 Effect of AS-IV on the expression of transdifferentiation proteins in renal tubular epithelial cells after UII intervention

**Fig 7:**
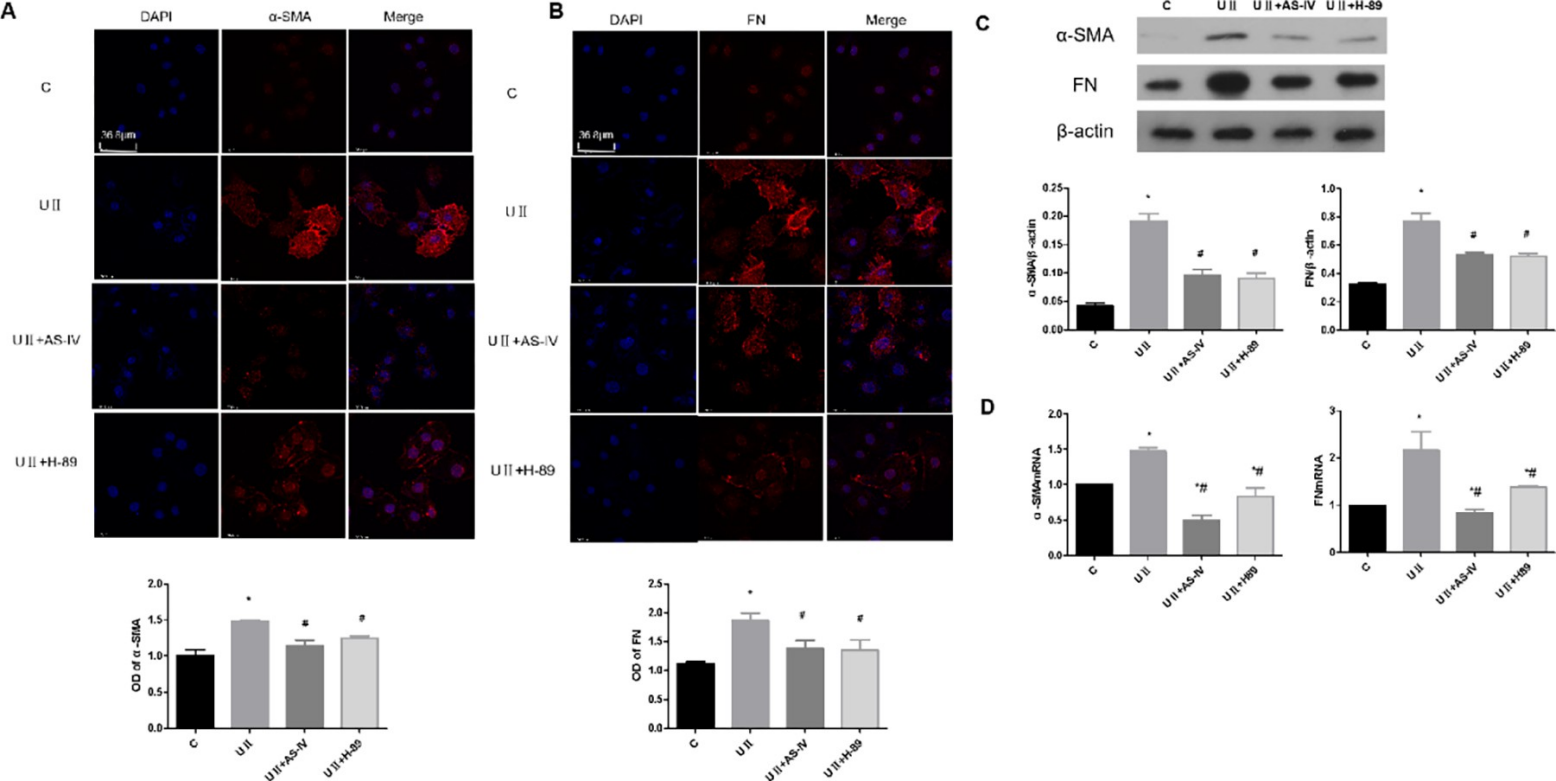
Effect of AS-IV on α-SMA and FN expression in renal tubular epithelial cells after UII intervention. (A) Effect of AS-IV on α-SMA immunofluorescence expression in renal tubular epithelial cells after UII intervention. (B) Effect of AS-IV on FN immunofluorescence expression in renal tubular epithelial cells after UII intervention. (C) Effect of AS-IV on α-SMA and FN protein expression in renal tubular epithelial cells after UII intervention. (D) Effect of AS-IV on mRNA expression of α-SMA and FN in renal tubular epithelial cells after UII intervention. * means *p*<0.05 compared with group C, # means *p*<0.05 compared with group UⅡ. (scale bar: 36.8 μm). The experiment was repeated three times.

Immunofluorescence results showed that UII intervention significantly enhanced α-SMA and FN fluorescence intensity in NRK-52E cells when compared to the control group (Fig 7A and 7B, *p*<0.05). However, after the intervention of AS-IV and PKA inhibitors, the fluorescence intensity of α-SMA and FN were significantly decreased (Fig 7A and 7B, *p* <0.05). Furthermore, western blot and RT-PCR results showed that UII intervention could induce the increased expression of α-SMA and FN proteins in NRK-52E cells (Fig 7C and 7D, *p*<0.05), while after the intervention of AS-IV and PKA inhibitors, the expressions of α-SMA and FN were decreased compared with those in UII group (Fig 7C and 7D, *p*<0.05). The results showed that UII could induce epithelial transdifferentiating (EMT) of NRK-52E cells, and AS-IV could inhibit UII-induced NRK-52E EMT.

## 4. Discussion

The global prevalence of CKD is 14.3%, and the prevalence of CKD in China is about 10.8% [23]. CKD has become a serious public health problem with high prevalence, and poor prognosis. Renal fibrosis is a complex dynamic process, whose features are the release of pro-fibrotic factors, the imbalance between synthesis and degradation of extracellular matrix, and excessive accumulation in renal interstitium. Renal microvascular disease leads to tissue ischemia and hypoxia [24]. The abnormal regulation of various cytokines and signaling pathways involved in the process of renal fibrosis is the core link in the progression of CKD to late-stage.

UII is the strongest vasoconstricting active peptide isolated from the pituitary gland at the tail of the spinal cord of bony fish [25]. G-protein-coupled receptor 14 (GPR14) is a specific receptor for UII [26]. In the kidney, the expression of GPR14 is most abundant in distal convoluted tubules, collecting duct epithelial cells, and glomerular capillary endothelial cells [27]. Some studies have found that UII combined with GPR14 can exert biological effects such as vasoconstriction, cell proliferation, and extracellular matrix expression and secretion [28–30], regulate inflammatory signaling pathways such as JAK2/STAT3 by inducing the production of pro-fibrotic factors such as TGF-β1 [31], and involved in the occurrence and development of renal fibrosis [32]. These studies suggested that UII is the key factor leading to renal fibrosis. Previous studies [33–35] by our research group showed that UII weakens the L-type calcium current of cardiomyocytes through the cyclic adenosine monophosphate/protein kinase A (cAMP/PKA) signal transduction pathway, resulting in promoting myocardial fibroblast proliferation and myocardial fibrosis. Further study [22] found that UII was involved in the occurrence and development of renal fibrosis in rats. UUO is a classic animal model of renal fibrosis. The results of this study showed that UUO rat renal pathology showed significant renal tubule dilatation, a large area of inflammatory cell infiltration in the renal interstitium, increased concentration of cAMP and UII in serum, and increased expression of pyroptosis index in renal tissue. The expression of pyroptosis and transdifferentiation indicators in renal tubular epithelial cells increased under the intervention of UII, suggesting that UII may be involved in the pathological process of renal fibrosis through the mechanism above.

The main mechanism of the G protein is to activate downstream adenylate cyclase (AC), causing the increase of cAMP, and cAMP as an important second messenger in the cell can activate PKA [36]. cAMP/PKA signaling pathway is one of the classical pathways of G protein-coupled receptor-mediated signal transduction [37]. PKA is a serine protein kinase, which acts on the phosphorylation of receptors, ion channels, transcription factors, and other proteins, and regulates the bioactive response and balance in cells [37]. After the activation of PKA by cAMP, the specific phosphate substrate, CAMP-Response element binding protein (CREB) is phosphorylated and specifically binds to the cAMP response element (CRE) in the nucleus, which regulates the transcription of downstream target genes and protein synthesis, thus producing biological effects [37]. Recent studies have shown that the cAMP/PKA signaling pathway is involved in regulating the fibrosis process of myocardial, lung, liver, kidney, and other organs [38,39]. Phosphorylation of CREB after activation of PKA by cAMP can competitively bind CREB-binding protein with Smad complex, thus down-regulating p-Smad2 level, inhibiting proliferation of fibroblasts and collagen synthesis [40], and thus improving renal fibrosis. Activation of the cAMP/PKA signaling pathway can regulate the expression of downstream factors such as TGF-β, induce mesenchymal transformation of renal tubular epithelial cells, and further promote the progression of renal fibrosis [39,41]. Inhibition of the PKA signaling pathway can directly block TGF-β1-induced glomerular sclerosis, thereby inhibiting renal fibrosis [42,43]. Inhibiting the activation of the cAMP/PKA/CREB signaling pathway and decreasing the expression of phenotype transforming protein α-SMA and extracellular matrix-related proteins LN and FN in renal tubular epithelial cells can delay the progression of renal fibrosis in diabetic nephropathy [44]. These studies suggest that Urotensin II and its regulated cAMP/PKA signaling pathway may be involved in the pathogenesis of renal fibrosis. The results of this study confirmed that Urotensin II intervention induces pyroptosis and epithelial cell transdifferentiation in renal tubular epithelial cells through activation of the cAMP/PKA signaling pathway.

Cell pyroptosis is an inflammatory and cytolysis of cell death induced by caspase-1, caspase-4/5, and caspase-11, and ultimately activates porin N-GSDMD, resulting in cell membrane destruction [45]. Recent studies have confirmed that cellular pyroptosis is involved in the disease progression of renal interstitial fibrosis [46,47]. Guo et al. [48] found that in the NLRP3 gene knockout UUO mice, activated caspase-1/IL-18/IL-1βwas significantly reduced and the glomerular damage and tubulointerstitial fibrosis were significantly reduced when compared with the wild-type mice. In patients with renal fibrosis, the expression levels of NLRP3 and Caspase-1 are significantly up-regulated [49], suggesting that the NLRP3 inflammasome may be activated and involved in regulating renal fibrosis. Recent studies have shown that the NLRP3/ASC/Caspase-1/IL-1β/IL-18 of cell pyroptosis signaling pathway-mediated inflammatory response promotes the pathological process of a variety of kidney diseases, and Caspase-11 activates Caspase-1 and stimulates the maturation of pro-IL-1, resulted in the promotion of renal fibrosis [50], suggesting that NLRP3-mediated inflammatory response and cellular pyroptosis may be involved in the occurrence and development of renal fibrosis. In recent years, several studies have confirmed that activation of the intracellular cAMP/PKA pathway can inhibit the activation of NLRP3 inflammasome and alleviate cell pyroptosis. Lee et al. [51] found that Ca^2+^ inhibited intracellular cAMP expression in macrophages and activated NLRP3 inflammasome through calcium-sensitive receptors. Hong et al. [52] found that ER stress can promote the activation of the cAMP/PKA pathway in type II alveolar epithelial cells, and the activation of the cAMP/PKA pathway can inhibit the activation of NLRP3 inflammasome induced by ER stress. A large number of studies have confirmed that activation of cAMP/PKA can inhibit the activation of NLRP3 inflammasome [53–56]. The results of this study confirmed that antagonizing the cAMP/PKA signaling pathway can inhibit the expression of pyroptosis-related indicators in renal tubule epithelial cells, suggesting that cAMP/PKA may act as a negative feedback regulator of NLRP3 inflammasome activation, thereby inhibiting pyroptosis of cells. However, the effects of UII antagonist (SB) and AS-IV on pyroptosis and the expression of NLRP3 and IL-1β were different, suggesting that the capacity of SB could improve the inflammatory damage of renal tubular epithelial cells more than that of AS-IV. Because there are multiple pathways of cell pyroptosis, this study only detected the classical Caspase-1 pathway, and AS-IV may also affect cell pyroptosis through other signaling pathways, thus, the effect is stronger than SB, and the specific mechanism needs to be further explored. Activation of NLRP3 can induce pyroptosis through the Caspse-1 pathway. The results of this study showed that various inflammatory cytokines released after cell membrane rupture during pyroptosis can also induce increased expression of α-SMA, FN, and Col-Ⅰ in peripheral renal tubule epithelium, resulting in EMT. AS-IV is one of the main active components of Astragalus, which has pharmacological properties such as anti-oxidative stress, immune regulation, anti-inflammatory, anti-apoptosis, and is widely used in the treatment of chronic kidney disease [57]. Previous studies of our research group have found [58] that AS-IV may improve cellular pyroptosis by down-regulating the expression of GSDMD and Caspase-1 in renal tissues of diabetic nephropathy. Another study [59] found that under the intervention of exosomes of mesenchymal stem cells mediated by AS-IV, the expressions of Caspase-1, GSDMD, and NLRP3 in human umbilical vein endothelial cells with high glucose-induced injury were reduced, indicating that AS-IV may inhibit cell pyroptosis. Our study demonstrated that AS-IV could improve renal fibrosis and reduce the levels of UII and cAMP in UUO rats, and *in vitro* studies showed that AS-IV could improve UII-induced renal tubular epithelial EMT. AS-IV was shown for the first time to ameliorate UII-induced pyroptosis of renal tubular epithelial cells. In summary, this study found that UII can induce pyroptosis and EMT in renal tubular epithelial cells through the cAMP/PKA signaling pathway, and AS-IV can improve UII-induced pyroptosis and EMT by inhibiting the cAMP/PKA signaling pathway, thus alleviating renal fibrosis. Our study provides a new theoretical and experimental basis for the scientific interpretation of AS-IV in the prevention and treatment of renal fibrosis.

## Supporting information

S1 Raw data (XLSX)

S1 Raw images (PDF)

## References

[1] Li J, Bao H, Zhang K, et al. MiR-542-3p drives renal fibrosis by targeting AGO1 in vivo and in vitro[J]. Life Sci, 2020,255:117845. 32470449 10.1016/j.lfs.2020.117845

[2] Jing H, Tang S, Lin S, et al. Adiponectin in renal fibrosis[J]. Aging (Albany NY), 2020,12(5):4660–4672. 32065783 10.18632/aging.102811 PMC7093169

[3] Lv W, Booz G W, Wang Y, et al. Inflammation and renal fibrosis: Recent developments on key signaling molecules as potential therapeutic targets[J]. Eur J Pharmacol, 2018,820:65–76. 29229532 10.1016/j.ejphar.2017.12.016 PMC6733417

[4] Menon M C, Ross M J. Epithelial-to-mesenchymal transition of tubular epithelial cells in renal fibrosis: a new twist on an old tale[J]. Kidney Int, 2016,89(2):263–266. 26806826 10.1016/j.kint.2015.12.025

[5] Lv W, Booz G W, Fan F, et al. Oxidative Stress and Renal Fibrosis: Recent Insights for the Development of Novel Therapeutic Strategies[J]. Front Physiol, 2018,9:105. 29503620 10.3389/fphys.2018.00105 PMC5820314

[6] Kim J, Jung KJ, Park KM. Reactive oxygen species differently regulate renal tubular epithelial and interstitial cell proliferation after ischemia and reperfusion injury [J]. Am J Physiol Renal Physiol, 2010, 298(5): F1118– F1129 20164154 10.1152/ajprenal.00701.2009

[7] Lovisa S, LeBleu VS, Tampe B, et al. Epithelial-to-mesenchymal transition induces cell cycle arrest and parenchymal damage in renal fibrosis[J]. Nat Med. 2015,21(9):998–1009. 26236991 10.1038/nm.3902 PMC4587560

[8] Chung YH, Huang GK, Kang CH, et al.MicroRNA-26a-5p Restoration Ameliorates Unilateral Ureteral Obstruction-Induced Renal Fibrosis in Mice Through Modulating TGF-β Signaling[J]. Lab Invest, 2023,103(7):100131. 36948295 10.1016/j.labinv.2023.100131

[9] Qi R C, Wang J Y, Jiang Y M, et al. Snai1-induced partial epithelialmesenchymal transition orchestrates p53-p21-mediated G2/M arrest in the progression of renal fibrosis via NF-κB-mediated inflammation[J]. Cell Death Dis, 2021,12:44. 33414422 10.1038/s41419-020-03322-y PMC7790819

[10] Zhao W, He C, Jiang J, et al. The role of discoid domain receptor 1 on renal tubular epithelial pyroptosis in diabetic nephropathy[J]. Korean J Physiol Pharmacol, 2022, 26(6):427–438. 36302618 10.4196/kjpp.2022.26.6.427 PMC9614395

[11] Zhang H, Wang Z. Effect and Regulation of the NLRP3 Inflammasome During Renal Fibrosis[J]. Front Cell Dev Biol, 2019,7:379. 32039201 10.3389/fcell.2019.00379 PMC6992891

[12] Pang XX, Bai Q, Wu F, Chen GJ, Zhang AH, Tang CS. Urotensin II Induces ER Stress and EMT and Increase Extracellular Matrix Production in Renal Tubular Epithelial Cell in Early Diabetic Mice[J]. Kidney Blood Press Res,2016,41(4):434–49. 27467277 10.1159/000443445

[13] Eyre H J, Speight T, Glazier J D, et al. Urotensin II in the development and progression of chronic kidney disease following (5/6) nephrectomy in the rat[J]. Exp Physiol, 2019,104(3):421–433. 30575177 10.1113/EP087366 PMC6492238

[14] Liang Y, Wu X, Xu M, et al. Urotensin II induces activation of NLRP3 and pyroptosis through calcineurin in cardiomyocytes[J]. Peptides, 2021,144:170609. 34242679 10.1016/j.peptides.2021.170609

[15] Pan Y J, Zhang M Z, He L H, et al. Expression of urotensin II is positively correlated with pyroptosis-related molecules in patients with severe preeclampsia[J]. Clin Exp Hypertens, 2021,43(3):295–304. 33371762 10.1080/10641963.2020.1867159

[16] Liu W, Han Q, Liu Q, et al. An investigation into the expression and mechanism of action of urotensin II in chronic pressure-overloaded rat hearts[J]. Mol Med Rep, 2015,12(5):6626–6634. 26323194 10.3892/mmr.2015.4244 PMC4626172

[17] Yoshikawa E, Matsui-Yuasa I, Huang X, et al. Mallotus furetianus extract protects against ethanol-induced liver injury via the activation of the cAMP-PKA pathway[J]. Food Sci Nutr, 2020,8(7):3936–3946. 32724654 10.1002/fsn3.1709 PMC7382178

[18] Guo C, Xie S, Chi Z, et al. Bile Acids Control Inflammation and Metabolic Disorder through Inhibition of NLRP3 Inflammasome[J]. Immunity, 2016,45(4):802–816. 27692610 10.1016/j.immuni.2016.09.008

[19] Pan H, Lin Y, Dou J, et al. Wedelolactone facilitates Ser/Thr phosphorylation of NLRP3 dependent on PKA signalling to block inflammasome activation and pyroptosis[J]. Cell Prolif, 2020,53(9):e12868. 32656909 10.1111/cpr.12868 PMC7507381

[20] Zhou X, Sun X, Gong X, et al. AS-IV from Astragalus membranaceus ameliorates renal interstitial fibrosis by inhibiting inflammation via TLR4/NF-small ka, CyrillicB in vivo and in vitro[J]. Int Immunopharmacol, 2017,42:18–24. 27855303 10.1016/j.intimp.2016.11.006

[21] Mao Q, Chen C, Liang H, et al. AS-IV inhibits excessive mesangial cell proliferation and renal fibrosis caused by diabetic nephropathy via modulation of the TGF-beta1/Smad/miR-192 signaling pathway[J]. Exp Ther Med, 2019,18(4):3053–3061. 31572545 10.3892/etm.2019.7887 PMC6755437

[22] 刘文媛,方敬爱,常沁涛,等.黄芪对压力超负荷大鼠肾脏UrotensinⅡ及胶原表达的影响[J].中国中西医结合肾病杂志,2013,14(12):1044–1046+10.

[23] Zhang L, Wang F, Wang L, et al. Prevalence of chronic kidney disease in China: a cross-sectional survey[J]. Lancet, 2012,379(9818):815–822. 22386035 10.1016/S0140-6736(12)60033-6

[24] Chen H, Fan Y, Jing H, et al. Emerging role of lncRNAs in renal fibrosis[J]. Arch Biochem Biophys, 2020,692:108530. 32768395 10.1016/j.abb.2020.108530

[25] Pearson D, Shively J E, Clark B R, et al. Urotensin II: a somatostatin-like peptide in the caudal neurosecretory system of fishes[J]. Proc Natl Acad Sci U S A, 1980,77(8):5021–5024. 6107911 10.1073/pnas.77.8.5021 PMC349982

[26] Ames R S, Sarau H M, Chambers J K, et al. Human urotensin-II is a potent vasoconstrictor and agonist for the orphan receptor GPR14[J]. Nature, 1999,401(6750):282–286. 10499587 10.1038/45809

[27] Balat A, Buyukcelik M. Urotensin-II: More Than a Mediator for Kidney[J]. Int J Nephrol, 2012,2012:249790. 23094156 10.1155/2012/249790 PMC3474241

[28] Soni H, Adebiyi A. Urotensin II-induced store-operated Ca(2+) entry contributes to glomerular mesangial cell proliferation and extracellular matrix protein production under high glucose conditions[J]. Sci Rep, 2017,7(1):18049. 29273760 10.1038/s41598-017-18143-x PMC5741753

[29] Cao Y K, Guo Q, Ma H J, et al. Microinjection of urotensin II into the rostral ventrolateral medulla increases sympathetic vasomotor tone via the GPR14/ERK pathway in rats[J]. Hypertens Res, 2020,43(8):765–771. 32385485 10.1038/s41440-020-0460-y

[30] Tian L, Fu P, Zhou M, et al. Role of urotensin II in advanced glycation end product-induced extracellular matrix synthesis in rat proximal tubular epithelial cells[J]. Int J Mol Med, 2016,38(6):1831–1838. 27840897 10.3892/ijmm.2016.2789

[31] Chen S, Wang Y, Wan Y. Urotensin II enhances transforming growth factor‑beta1 expression and secretion in the kidney during aristolochic acid nephropathy[J]. Mol Med Rep, 2017,16(5):6904–6909. 28901401 10.3892/mmr.2017.7424

[32] Wang T, Xie Y Q, Miao G X, et al. Urotensin receptor antagonist urantide improves atherosclerosis-related kidney injury by inhibiting JAK2/STAT3 signaling pathway in rats[J]. Life Sci, 2020,247:117421. 32061865 10.1016/j.lfs.2020.117421

[33] Liu WY, Liu QH, Han QH, et al. Effect of urotensin II on apoptosis of H9c2 cardiomyocytes and the underlying mechanisms. Int J Clin Exp Med,2017,10(8):12012–12017.

[34] Liu WY, Han Q, Liu Q, et al. An investigation into the expression and mechanism of action of urotensin II in chronic pressure-overloaded rat hearts. Mol Med Rep,2015,12(5):6626–6634. doi: 10.3892/mmr.2015.4244 26323194 PMC4626172

[35] Xu J, Han Q, Shi H, et al. Role of PKA in the process of neonatal cardiomyocyte hypertrophy induced by urotensin II[J]. Int J Mol Med, 2017,40(2):499–504. 28656205 10.3892/ijmm.2017.3038

[36] Edra London, Stratakis Constantine A. The regulation of PKA signaling in obesity and in the maintenance of metabolic health.[J].Pharmacol Ther, 2022, 237: 108113. doi: 10.1016/j.pharmthera.2022.108113 35051439

[37] Deb Dilip K, Bao Riyue, Li Yan Chun. Critical role of the cAMP-PKA pathway in hyperglycemia-induced epigenetic activation of fibrogenic program in the kidney.[J].FASEB J, 2017, 31: 2065–2075. 28148567 10.1096/fj.201601116R PMC5388553

[38] Delaunay M, Osman H, Kaiser S, et al. The Role of Cyclic AMP Signaling in Cardiac Fibrosis[J]. Cells, 2019,9(1). 10.3390/cells9010069 PMC7016856 31888098

[39] Deb D K, Bao R, Li Y C. Critical role of the cAMP-PKA pathway in hyperglycemia-induced epigenetic activation of fibrogenic program in the kidney[J]. FASEB J, 2017,31(5):2065–2075. 28148567 10.1096/fj.201601116R PMC5388553

[40] Liu Y, Xu H, Geng Y, et al. Dibutyryl-cAMP attenuates pulmonary fibrosis by blocking myofibroblast differentiation via PKA/CREB/CBP signaling in rats with silicosis[J]. Respir Res, 2017,18(1):38. 28222740 10.1186/s12931-017-0523-z PMC5320641

[41] Schinner E, Wetzl V, Schlossmann J. Cyclic nucleotide signalling in kidney fibrosis[J]. Int J Mol Sci, 2015,16(2):2320–2351. 25622251 10.3390/ijms16022320 PMC4346839

[42] Ding H, Bai F, Cao H, et al. PDE/cAMP/Epac/C/EBP-beta Signaling Cascade Regulates Mitochondria Biogenesis of Tubular Epithelial Cells in Renal Fibrosis[J]. Antioxid Redox Signal, 2018,29(7):637–652. 29216750 10.1089/ars.2017.7041

[43] Weng L, Wang W, Su X, et al. The Effect of cAMP-PKA Activation on TGF-beta1-Induced Profibrotic Signaling[J]. Cell Physiol Biochem, 2015,36(5):1911–1927. 26202352 10.1159/000430160

[44] 黄倩,张素萍,施子禄.人参多糖通过cAMP/PKA/CREB信号通路抗糖尿病肾病肾纤维化作用机制研究.中国药理学通报, 2018,34(05):695–701.

[45] Sharma D, Kanneganti T D. The cell biology of inflammasomes: Mechanisms of inflammasome activation and regulation[J]. J Cell Biol, 2016,213(6):617–629. 27325789 10.1083/jcb.201602089 PMC4915194

[46] 吴燕升, 高建东.细胞焦亡在肾脏炎性损伤中的作用研究进展[J].基础医学与临床, 2017,37(10):1470.

[47] 刘建铭, 曹灵. 细胞焦亡信号转导机制及其在肾脏疾病发病中作用的研究进展[J].山东医药, 2018,58(30):92.

[48] Guo H, Bi X, Zhou P, et al. NLRP3 Deficiency Attenuates Renal Fibrosis and Ameliorates Mitochondrial Dysfunction in a Mouse Unilateral Ureteral Obstruction Model of Chronic Kidney Disease[J]. Mediators Inflamm, 2017,2017:8316560. 28348462 10.1155/2017/8316560 PMC5350413

[49] Ke B, Shen W, Fang X, et al. The NLPR3 inflammasome and obesity-related kidney disease[J]. J Cell Mol Med, 2018,22(1):16–24. 28857469 10.1111/jcmm.13333 PMC5742686

[50] Miao N J, Xie H Y, Xu D, et al. Caspase-11 promotes renal fibrosis by stimulating IL-1beta maturation via activating caspase-1[J]. Acta Pharmacol Sin, 2019,40(6):790–800. 30382182 10.1038/s41401-018-0177-5 PMC6786359

[51] Lee G S, Subramanian N, Kim A I, et al. The calcium-sensing receptor regulates the NLRP3 inflammasome through Ca2+ and cAMP[J]. Nature, 2012,492(7427):123–127. 23143333 10.1038/nature11588 PMC4175565

[52] Hong Q, Zhang Y, Lin W, et al. Negative Feedback of the cAMP/PKA Pathway Regulates the Effects of Endoplasmic Reticulum Stress-Induced NLRP3 Inflammasome Activation on Type II Alveolar Epithelial Cell Pyroptosis as a Novel Mechanism of BLM-Induced Pulmonary Fibrosis[J]. J Immunol Res, 2022,2022:2291877. 36033388 10.1155/2022/2291877 PMC9410862

[53] Yan Y, Jiang W, Liu L, et al. Dopamine controls systemic inflammation through inhibition of NLRP3 inflammasome[J]. Cell, 2015,160(1–2):62–73. 25594175 10.1016/j.cell.2014.11.047

[54] Chen Y, Le TH, Du Q, et al. Genistein protects against DSS-induced colitis by inhibiting NLRP3 inflammasome via TGR5-cAMP signaling[J]. Int Immunopharmacol, 2019,71:144–154. 30901677 10.1016/j.intimp.2019.01.021

[55] Chen R, Zeng L, Zhu S, et al. cAMP metabolism controls caspase-11 inflammasome activation and pyroptosis in sepsis[J]. Sci Adv, 2019,5(5):eaav5562. 31131320 10.1126/sciadv.aav5562 PMC6531004

[56] Sokolowska M, Chen L Y, Liu Y, et al. Prostaglandin E2 Inhibits NLRP3 Inflammasome Activation through EP4 Receptor and Intracellular Cyclic AMP in Human Macrophages[J]. J Immunol, 2015,194(11):5472–5487. 25917098 10.4049/jimmunol.1401343 PMC4433768

[57] 李冀, 王田, 付强, 等.黄芪甲苷对肾脏的保护作用研究进展[J].吉林中医药, 2022,42(10):1214–1218.

[58] Zhang M, Liu W, Liu Y, et al. AS-IV Inhibited Podocyte Pyroptosis in Diabetic Kidney Disease by Regulating SIRT6/HIF-1α Axis[J].DNA Cell Biol. 2023,42(10):594–607. 37751175 10.1089/dna.2023.0102

[59] 熊武, 谭梅鑫, 陈姿霖, 等.黄芪甲苷介导间充质干细胞外泌体对高糖诱导损伤的内皮细胞生物学功能和细胞焦亡的影响[J].中国医师杂志, 2021,23(12):1769–1773,1781.

