# Supplementary material for: Astragaloside IV alleviates renal fibrosis by inhibiting renal tubular epithelial cell pyroptosis induced by urotensin II through regulating the cAMP/PKA signaling pathway
Source: PLoS One. 2024 May 31;19(5):e0304365. doi: 10.1371/journal.pone.0304365 (PMC11142519; doi:10.1371/journal.pone.0304365)
Supplement: S1 Raw images — (PDF) [file pone.0304365.s002.pdf]

Astragaloside IV alleviates renal fibrosis by inhibiting renal tubular epithelial cell pyroptosis induced by urotensin II through regulating the cAMP/PKA signaling pathway

Lin Zhang[ab], Wenyuan Liu[c], Sufen Li[a], Jinjing Wang[a], Dalin Sun[a], Hui Li[c], Ziyuan Zhang[c], Yaling Hu[c], Jingai Fang*[c]

Short title: AS-IV improves renal fibrosis

[a]Shanxi Medical University, 56 Xinjian South Road, Taiyuan, Shanxi Province 030001, China

[b]Department of prevention care, Cardiovascular Hospital of Shanxi Medical University, 18 Yifen Street, Taiyuan, Shanxi Province 030001, China

[c]Department of Nephrology, First Hospital of Shanxi Medical University, 85 Jiefang South Road, Taiyuan, Shanxi Province 030001, China

*Correspondence: Jingai Fang

First Hospital of Shanxi Medical University,

85 Jiefangnan Road, Taiyuan, Shanxi 030001, China

Mail address: jingaifang2017003@126.com

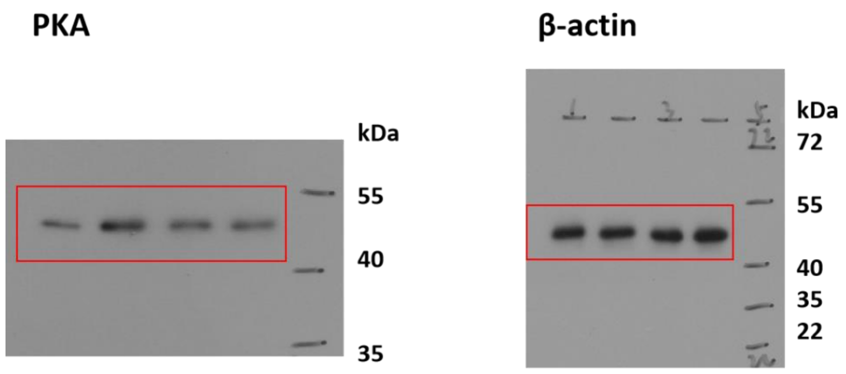

Fig. S1：Original western blots of Fig.5.

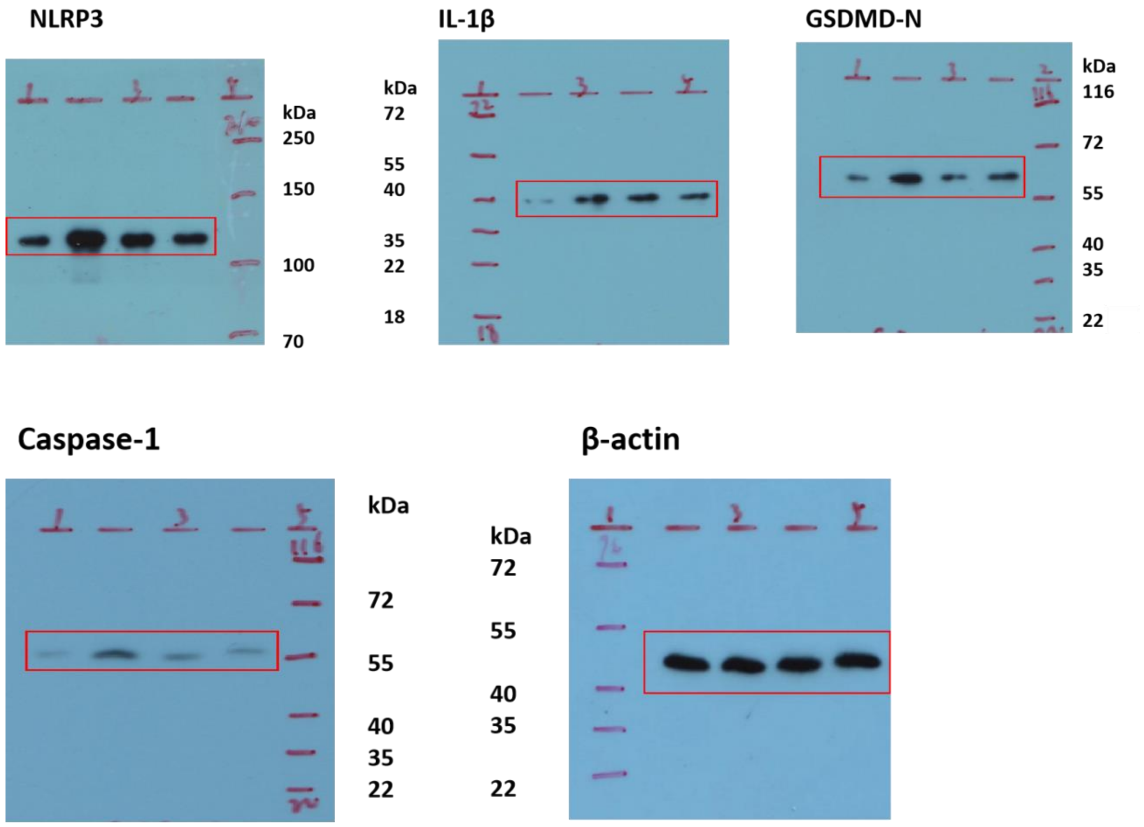

Fig.2： Original western blots of Fig.6A.

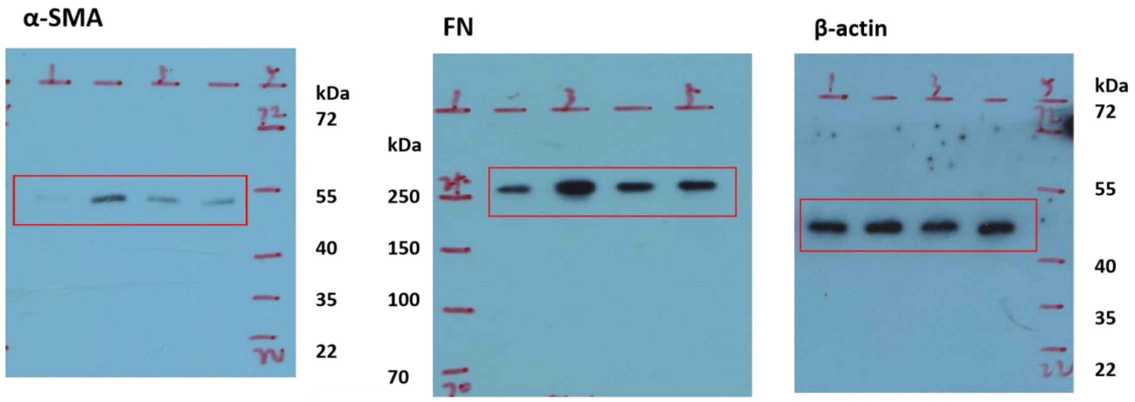

Fig.3： Original western blots of Fig.7C.
